# The full-genome characterization and phylogenetic analysis of bovine herpesvirus type 1.2 isolated in China

**DOI:** 10.3389/fmicb.2022.1033008

**Published:** 2022-11-01

**Authors:** Weiqiang Guo, Jia Xie, Jingyi Liu, Hongjun Chen, Yong-Sam Jung

**Affiliations:** ^1^MOE Joint International Research Laboratory of Animal Health and Food Safety, College of Veterinary Medicine, Nanjing Agricultural University, Nanjing, Jiangsu, China; ^2^Shanghai Veterinary Research Institute, Chinese Academy of Agricultural Sciences, Shanghai, China

**Keywords:** bovine herpesvirus type 1.2, isolation, full genome, phylogenetic analysis, BHV-1.2

## Abstract

Bovine herpesvirus type 1 (BHV-1) causes bovine respiratory disease that poses a significant threat to the cattle industry. The prevalence of BHV-1 has recently increased in China. However, the lack of information about the prevalent isolates limits the control of the disease. In this study, a novel strain of BHV-1 was isolated from nasal swabs of Holstein cows in 2020 in China, designated as BHV SHJS. The genome of BHV strain SHJS is 135, 102 bp in length and highly similar to strain SP1777 (KM258883.1) with an identity of 99.64%. Mutations, insertions, or deletions mainly occur in UL27, UL44, and US8, etc., relative to the different genomic coordinates. Phylogenetic tree of UL44 (gC) showed that BHV strain SHJS belongs to BHV-1.2b cluster. The result showed that the strain had a different evolutionary origin from those prevalent in China. This study will enrich our knowledge regarding BHV outbreak strains in China and contribute to the prevention and pathogenic studies of BHV-1.2.

## Introduction

Bovine herpesvirus type 1 (BHV-1) was a key pathogen causing a contagious bovine respiratory disease or abortion that poses a threat and significant economic losses in cattle industry (Brake and Studdert, 1985; Hodgson et al., 2005; Jones and Chowdhury, 2007). BHV-1 belongs to the family *Herpesviridae*, subfamily *Alphaherpesvirinae* (Nandi et al., 2009). It was divided into BHV-1.1 and 1.2 types, which was further classified into two gene subtypes: BHV 1.2a and 1.2b (Metzler et al., 1985; Zhou et al., 2020).

Bovine herpesvirus type 1 exhibits a double-stranded DNA of approximately 135 kb in size (Engels et al., 1987). The BHV-1 genome contains a unique long sequence region (UL), a unique short sequences region (US), an internal repeat sequences region (IR) between UL and US, and an inverted terminal repeat sequences region (TR) (Leung-Tack, et al., 1994; d'Offay et al., 2016). It encodes about 73 open reading frames (ORFs) (Engels et al., 1986). Glycoprotein B (gB), glycoprotein C (gC), and glycoprotein D (gD) encoded by UL27, UL44, and US6, respectively, were the major attachment proteins, which could induce a high level of neutralizing antibodies against BHV-1 (Babiuk et al., 1987; Van Drunen Littel-van den Hurk et al., 1990; Liang et al., 1991). Inactivated or attenuated vaccines are used against BHV-1 conventionally (Deshpande et al., 2002; O'Toole et al., 2012; Marawan et al., 2021). However, they only partially protect from wild-type BHV-1 infection (O'Toole et al., 2014; Fulton et al., 2015, 2016).

In China, the prevalence of BHV-1 presented fluctuations of 5 to 68.7% from 2012 to 2017 in cattle (Zhong et al., 2012; Pi et al., 2014; Mo and Liu, 2017). A meta-analysis showed the prevalence of BHV infection even increased up to 40% in China (Chen et al., 2018). To date, information about the prevalent isolates, and biological characterization of BHV-1 strains were scarce in China. Therefore, isolation of epidemic strains, analysis of genome of isolates on evolutionary status, and exploration of biological characterization were very meaningful to enrich our knowledge regarding BHV outbreak strains.

In 2020, a local farm of Holstein cows developed severe respiratory inflammation and abortion symptoms in Shanghai, China. We suspected that those cows might be infected with BHV-1. In this study, a novel strain of BHV-1.2b was isolated from the nasal swabs. The genome of the isolate was sequenced and the biological characteristics of the virus were systemically investigated. This study will contribute to investigating the evolution of BHV-1.2b and aid in its future prevention or management.

## Materials and methods

### Cells and virus isolation

Madin-Darby bovine kidney cells (MDBK) were purchased from the American Type Culture Collection and stored in the laboratory. Cells were incubated using Dulbecco’s Modified Eagle’s Medium (DMEM) (BI, China) with 8% fetal bovine serum (FBS) (PAN Biotech, Germany) at 37°C in a 5% CO_2_ incubator. Thirty-three nasal swabs were collected from Holstein cows from a bovine farm in Jinshan District, Shanghai, China, in 2020. BHV-1 US7 positive samples were homogenized with DMEM and centrifuged using 6, 000 × *g* for 5 min. The supernatant was filtered by a filter unit and then inoculated onto MDBK cell monolayers grown for virus isolation by monitoring cytopathic effects (CPEs).

### Polymerase chain reaction

DNA was extracted from nasal swabs and the supernatant of the infected cells using the Ezup column viral DNA kit (Sangon Biotech, China). Specific primers were designed to amplify BHV-1 US7, US8, UL48, and UL49 genes (Supplementary Table S1). PCR reactions were amplified using DNA polymerase (Vazyme Biotech, China) under the conditions: 95°C for 3 min, 35 cycles at 95°C for 15 s, 68°C for 1 min, and 72°C for 2 min (Waters and Shapter, 2014).

### Plaque formation assay

MDBK cells were infected with the virus of a serial 10-fold dilution. Following 2-h incubation, the cells were washed thrice using PBS buffer. Then, the infected cells were overlaid using 1% agarose (Invitrogen, United States) that was low-melting point and supplemented with 2% FBS. Then, the cells overlaid with agarose were placed 4°C for 5 min. Next, the cells were incubated at 37°C and observed by plaque formation assay. Fifty plaques were randomly selected for virus passage and purification (Moe et al., 1981). The diameter was measured using the software of Image J (National Institutes of Health).

### Growth curve and virus titration

The growth kinetics of the virus was determined by infecting MDBK cell monolayers with the virus at a multiplicity of infection (MOI = 1) for 2 h post-infection (hpi). Then, cells were washed thrice with PBS and replaced with DMEM with 2% FBS. The cells and supernatant of infection were collected at different time points (6, 12, 24, 36, and 48 hpi). Following three freeze–thaw cycles at −80°C, the pellets and supernatants were prepared at centrifugation of 12, 000 × *g* for 3 min. The TCID_50_ of these samples were determined using 96-well plates coated with MDBK cell monolayers. Cells were infected with the virus of a serial 10-fold dilutions. The CPEs of the cells were observed daily. The TCID_50_ were determined according to the Reed and Muench method (Reed and Muench, 1938).

### Genomic sequencing and phylogenetic analysis

Viral DNA was extracted using Viral DNA kit (Omega, United States) from the supernatant of infected cells referring to the instructions. The full-genome sequence was obtained using the second-generation sequencing platform of the GenScript company. The full-genome sequence was compared with the published BHV-1 complete genomes in the NCBI database. The full genome of the isolated BHV-1 strain was analyzed using the viral genome with the highest homology as a reference. Phylogenetic analyses based on BHV-1 UL44 (gC) and UL27 (gB) sequences were performed using MEGA software (version 7.0.26). The reference sequences of UL44 (gC) and UL27 (gB) genes retrieved from GenBank for phylogenetic analysis are listed in Table 1.

**Table 1:** The list of reference sequences of BHV strains.

Isolates	Country	Host or vaccine	Accession number	Gene analyzed	Collection date
HL4_2019	China	Beef cattle	MT179811.1	UL44	2019
JL1_2016	China	Beef cattle	MT179813.1	UL44	2016
LN2_2019	China	Beef cattle	MT179816.1	UL44	2019
NM4_2019	China	Beef cattle	MT179820.1	UL44	2019
IBRV/2018/SMU6352	China	Cattle	MK654723.1	UL27	2018
HL3_2018	China	Dairy cow	MT179810.1	UL44	2018
LN1_2018	China	Beef cattle	MT179815.1	UL44	2018
NM3_2017	China	Dairy cow	MT179819.1	UL44	2017
XT-IBRV	China	Bovine	MF287966.1	UL27	2017
JL2_2017	China	Beef cattle	MT179814.1	UL44	2017
IBRV-4 T3-1	China	Bos taurus	KY348790.1	UL27	2016
HL1_2016	China	Dairy cow	MT179808.1	UL44	2016
NM1_2016	China	Beef cattle	MT179817.1	UL44	2016
NM2_2016	China	Dairy cow	MT179818.1	UL44	2016
PA1	United States	Bos taurus	MG407790.1	Complete genome	2015
PA2	United States	Bos taurus	MG407791.1	Complete genome	2015
PA3	United States	Bos taurus	MG407792.1	Complete genome	2015
C35	United States	Bos taurus	MH598937.1	UL27	2014
Abu-Hammad	Egypt	Cattle	KJ652519.1	UL27	2013
Abu-Hammad 2	Egypt	Cattle	KJ652520.1	UL27	2013
MN15	United States	–	MG407789.1	UL44/UL27	2013
TSV-2 Nasal MLV	United States	Vaccine	MH724209.1	Complete genome	2013
Nasalgen IP MLV	United States	Vaccine	MH724210.1	Complete genome	2013
IBRV-XJ	China	Cow	JN106448.1	UL27	2009
Uruguay T4	Uruguay	Bos taurus	JN173251.1	UL44	2012
MN14	United States	–	MG407788.1	UL44/UL27	2012
NVSL challenge 97–11	United States	Bos taurus	JX898220.1	Complete genome	2012
MN13	United States	Cattle	MG407787.1	UL27	2011
MN4	United States	Cattle	KJ652516.1	UL44/UL27	2010
MN6	United States	Cattle	MG407780.1	UL44/UL27	2010
MN7	United States	Cattle	MG407781.1	UL27	2010
MN8	United States	Cattle	MG407782.1	UL27	2010
MN9	United States	Cattle	MG407783.1	UL27	2010
MN10	United States	Cattle	MG407784.1	UL27	2010
MN12	United States	Cattle	MG407786.1	UL27	2010
IBRV-DX	China	Cow	JN106444.1	UL27	2009
IBRV-DM	China	Cow	JN106443.1	UL27	2009
IBRV-SZ	China	Cow	JN106446.1	UL27	2009
IBRV-LJ	China	Cow	JN106445.1	UL27	2009
IBRV-XE	China	Cow	JN106447.1	UL27	2009
SP1777	United States	–	KM258883.1	Complete genome	2009
MN5	United States	Cattle	MG407779.1	Complete genome	2009
MN2	United States	Cattle	KJ652514.1	UL44/UL27	2008
MN4	United States	Cattle	MG407778.1	UL44/UL27	2008
SV97-07	Brazil	Bos taurus	JN173248.1	UL44	2007
SV102-07	Brazil	Bos taurus	JN173249.1	UL44	2007
SV71-07E	Brazil	Bos taurus	JN173246.1	UL44	2007
NM06	China	Milch cow	JN787952.1	UL27	2006
COOPER	United States	–	DQ173733.1	UL44	2005
MN1	United States	Cattle	KJ652513.1	Complete genome	2004
C14	United States	Bos taurus	MH598936.1	UL27	2003
B589	Australia	Bovine	KM258881.1	Complete genome	2001
SV507/99	Brazil	Bos taurus	NC_005261.3	UL44	1999
Jura	Switzerland	–	AJ004801.1	UL44/UL27	1992
C1Z FZR	Germany	Bos taurus	MT862163.1	UL44	1986
SM023	United States	Bos taurus	KM258882.1	Complete genome	1986
Riems 8/85	Germany	Bos taurus	MT862164.1	UL44	1985
C28	United States	Bos taurus	MH598938.1	UL27	1981
C45	United States	Bos taurus	MH791339.1	UL27	1979
C46	United States	Bos taurus	MH791340.1	UL27	1979
C42	United States	Bos taurus	MH791336.1	UL27	1978
C43	United States	Bos taurus	MH791339.1	UL27	1978
C44	United States	Bos taurus	MH791338.1	UL27	1978
C47	United States	Bos taurus	MH791341.1	UL27	1978
216 II	India	Bos indicus	KY215944	Complete genome	1976
COOPER	United States	–	KU198480.1	Complete genome	1966
K22	United States	Bos taurus	KM258880.1	Complete genome	1966
ATCC:VR-188	United States	Bos taurus	MF421714.1	UL44	1956
Los Angeles 1956	United States	Bos taurus	MF421714.1	UL44	-
EVI14 2005	Brazil	–	DQ173739.1	UL44	
UY1999	Uruguay	–	DQ173735.1	UL44	2005
2010/2	United States	Cattle	KJ652525.1	UL44	2010
Arsenal IBR MLV	United States	Vaccine	MH724202.1	Complete genome	–
Titanium IBR MLV	United States	Vaccine	MH724203.1	Complete genome	–
Express1IBR MLV	United States	Vaccine	MH724204.1	Complete genome	–
Pyramid IBR MLV	United States	Vaccine	MH724205.1	Complete genome	–
Vista IBR MLV	United States	Vaccine	MH724206.1	Complete genome	–
Bovi-Shield IBR MLV	United States	Vaccine	MH724207.1	Complete genome	–
Bovi-Shield Gold FP5 MLV	United States	Vaccine	MH724208.1	Complete genome	–

§, no presentation or unknown.

### Alignment and prediction of structure

The amino acid sequences were analyzed by alignment online.^1^ Protein-encoded UL42 and UL46 secondary structures were predicted by PredictProtein.^2^ Protein domains were predicted by SMART.^3^

### Western-blotting analysis

The cells extracts were processed using Sodium Dodecyl Sulfate – Polyacrylamide Gel Electrophoresis (SDS-PAGE). At room temperature, proteins were electrophoresed to polyvinylidene fluoride (PVDF) membranes (Pall Corp, China) and blocked with 5% non-fat milk in PBS for 1 h. The membranes were incubated overnight at 4°C with BHV-VP22 polysera (prepared in our lab) and β-actin antibody (CST, United States). Then, membranes were washed thrice in PBST and incubated with secondary antibody (Sigma, United States). Membrane signals were detected using a Pierce™ SuperSignal West Pico Chemiluminescent Substrate using a Chemiluminescence Immunoassay image analysis system (Tanon 5,200, China).

### Immunofluorescence assay

Viral replication was investigated on coverslips with MDBK cell monolayer at 6, 12, 18, 24, 36, and 48 hpi. Monolayers were incubated and then fixed with 4% formaldehyde at 37°C for 35 min. Then, the cells were washed with PBS buffer and blocked with PBS with 5% milk of non-fat at 37°C for 30 min. Cells were washed with PBS buffer and incubated with BHV-VP22 polysera (1,500 dilution) for 45 min, followed by washing with PBS and staining with 4′, 6-diamidino-2-phenylindole (DAPI). Images were captured under an ECHO fluorescence microscope (RVL-100-g, United States).

### Statistical analysis

Data were collated and presented as mean ± standard deviation (SD). Differences of the groups were determined by *t*-test with GraphPad Prism software (version 7.0). A *value of p* smaller than 0.05 was considered statistically significant (*p < 0.05; **p < 0.01; ***p < 0.001).

## Results

### Isolation and biological characteristics of isolated virus

**Figure 1:**
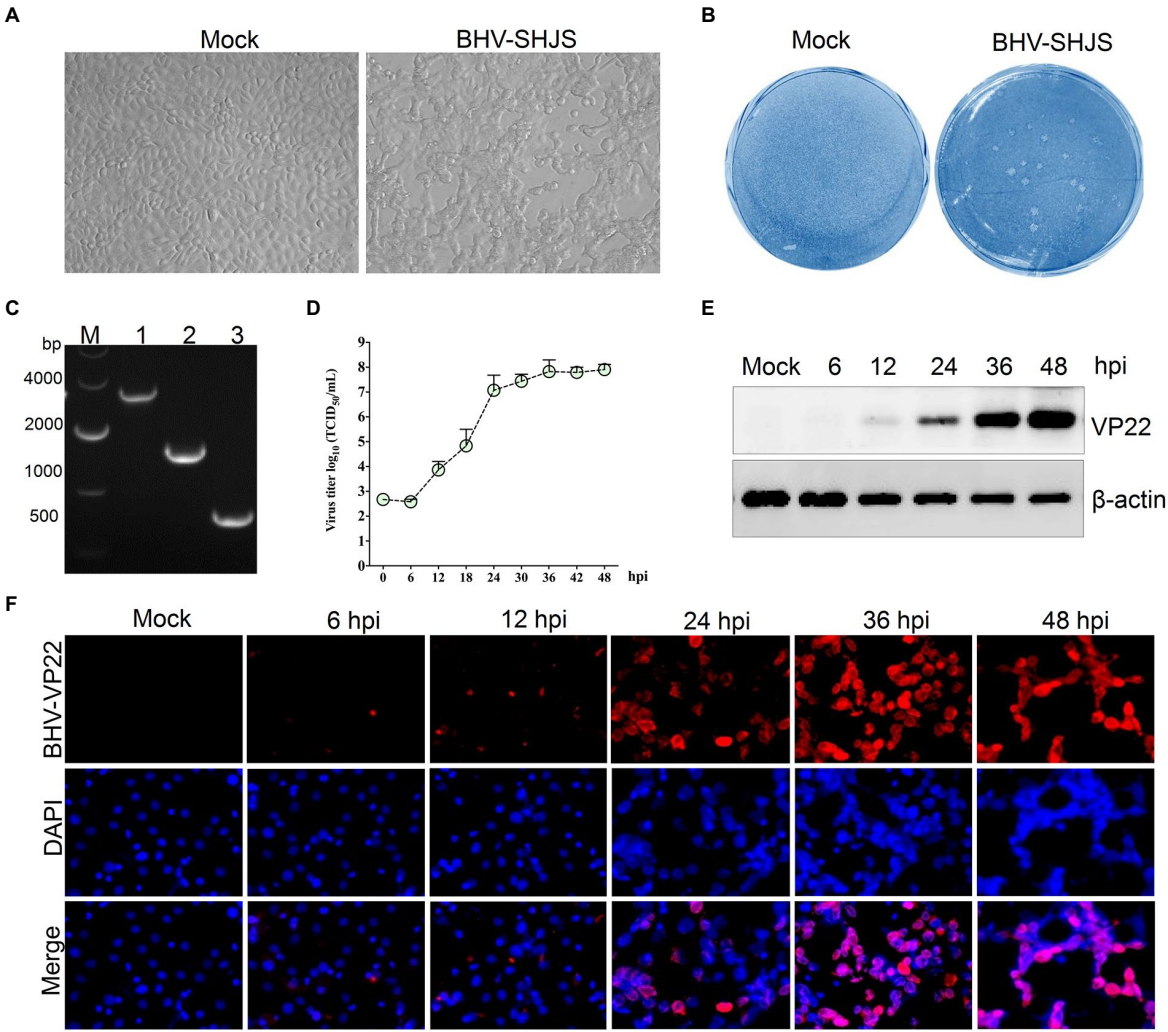
Isolation, identification, and biological characterization of the isolated BHV SHJS strain. **(A)** Isolation of virus on MDBK cells. Virus was isolated from the nasal swab. CPE was observed on BHV SHJS-infected and mock cells. **(B)** Plaque assay. MDBK cells were infected with the isolated virus. **(C)** Amplification of the conserved gene in infected cells. The fragments were amplified from extracted DNA from the supernatant of infected cells using specific primers for BHV-1 US7/US8 (lane 1), UL48 (lane 2), UL49 (lane 3). **(D)** Growth curves of BHV SHJS in MDBK cells. Cells were infected with BHV SHJS (MOI = 1). The TCID_50_ values were determined by the Reed and Muench method. Dynamic changes of viral infection were verified by western blotting **(E)** and IFA **(F)**. The cells infected with the virus (MOI = 1) were incubated and recognized using VP22 polysera.

Four samples were identified as positive-BHV from 33 nasal swabs (Supplementary Figure S1). Positive samples were inoculated on MDBK cells to obtain the virus. Only one sample showed visible CPEs with round and enlarged cells shrunk or detached at 36 hpi (Figure 1A). After three purification cycles, 1 to 2 mm diameter plaques were formed in infected cells compared with the mock group (Figure 1B). Three fragments were amplified from the infected cells by PCR (Figure 1C). Further sequence analysis revealed that the three amplified fragments showed high similarity to BHV-1 US7/US8 (3,156 bp), UL48 (1,524 bp), and UL49 (776 bp) (Supplementary Figure S2), respectively. The data suggested that the isolated virus was BHV-1.2b, referred to as BHV SHJS. To investigate the biological characteristics of isolated strain, the growth curve was determined on cells infected with the purified BHV SHJS (MOI = 1) at 6, 12, 18, 24, 30, 36, 42, and 48 hpi. The results showed that the virus titer was 8.02 × 10^7^ TCID_50_/mL at 48 hpi (Figure 1D). Western blotting and IFA analysis showed that virus infection could be detected at 12 hpi and 6 hpi, respectively (Figures 1E,F).

### Characterization of BHV SHJS genome

**Figure 2:**
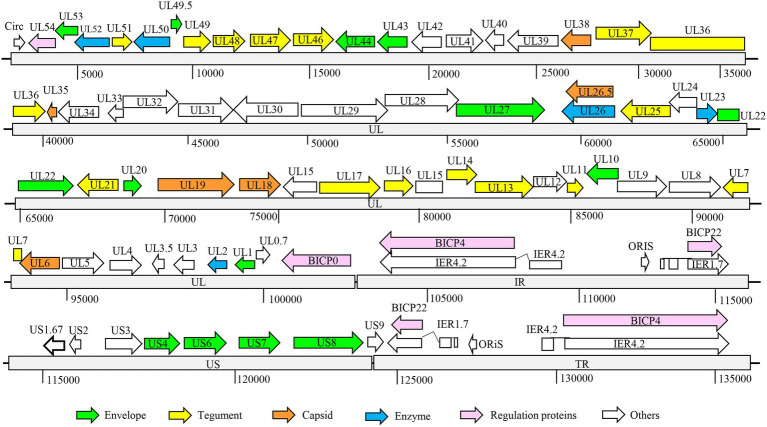
Organization of BHV SHJS full-genome map. Genome of BHV SHJS contains unique long sequence region (UL) and unique short (US) sequences region. An internal repeat sequence region exists between the UL and US region, called the IR region. Inverted repeat sequences, called the TR region, are present at the end of the genome US region.

The full genome of BHV strain SHJS was sequenced by second-generation sequencing. After assembly, the genome of a 135, 102 bp size of BHV strain SHJS was obtained. The average coverage depth of the assembled sequences was 3332.6×. The genome of strain SHJS has been submitted to NCBI (GenBank accession number: OP035381). Although BHV-1.2b is prevalent in China, no full-genome map of the BHV-1.2b isolates has been reported. We found that the BHV SHJS genome was collinear with BHV-1 K22 (KM258880.1) genome (d'Offay et al., 2016). The full genome of the BHV strain SHJS virus included a unique long sequence region (UL), a unique short sequences region (US), an inverted repeat sequences region (IR), and an inverted terminal repeat sequences region (TR). Sequence analysis showed that the BHV SHJS genome encodes 72 ORFs. Among them, 32 ORFs encode viral structure proteins, five ORFs encode related enzymes, and six ORFs encode regulatory function-related proteins. These are marked in different colors in the map of BHV SHJS genome (Figure 2). The position information of the genes revealed in the Supplementary Table S2.

### Analysis of BHV SHJS genome

**Figure 3:**
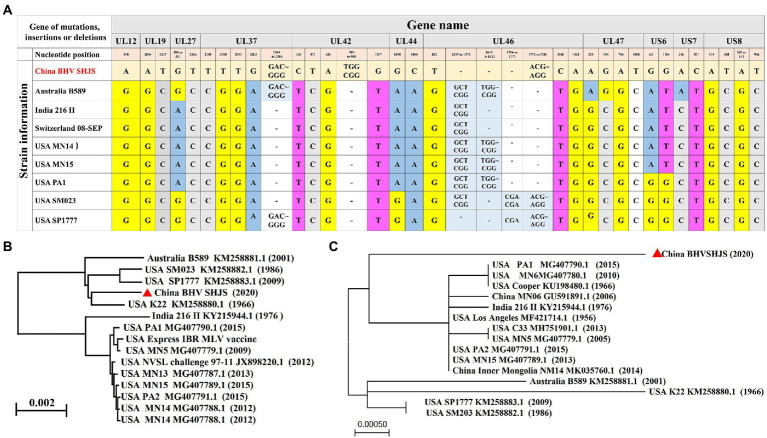
Phylogenetic analysis of the complete genome and US8 (gE) sequence. **(A)** Analysis of the complete genome sequence for BHV SHJS. **(B)** Phylogenetic analysis of full-genome sequence of BHV SHJS determined using the Neighbor Joining method in molecular evolutionary genetics on NCBI. **(C)** Phylogenetic analysis of US8 (gE) sequence using Maximum likehood method by MEGA software.

BHV strain SHJS genome shared 98.57 to 99.64% nucleotide identity with 25 BHV-1 strains (including American, Australian, and Indian strains) available in GenBank, and the highest identity was shown with strain SP1777 (KM258883.1) with a homolog of 99.64%. Genome sequence alignment showed that mutations, insertions, or deletions were observed in BHV SHJS UL12, UL19, UL27, UL37, UL42, UL44, UL46, UL47, US6, US7, and US8 genes compared to its different genomic coordinates (Figure 3A).

Amino acid alignment of these sequences showed that 41 Val (V) and 204 Gly (G) were mutated to Ile (I) and Ser (S) in BHV strain SHJS US8 (gE), respectively (Supplementary Figure S3A). Two insertions, including 333 Ser (S) and 334 Ala (A), and one mutation at 369 H (His) that mutated to Q (Gln) were observed in BHV SHJS UL42 (Supplementary Figure S3B). Two deletion sites at 456 to 457, 552 to 571, and 648 to 653, two insertion regions located at 611 to 614 and 649 to 656, and one mutation site at 514 (Phe) that mutated to C (Cys) were found in the sequence of BHV SHJS UL46 (Supplementary Figure S3C). The data suggested that the genomic differences of BHV SHJS compared with that of the previously reported BHV strains occur at US8, UL42, and UL46 genes.

### Phylogenetic analysis of the genome and US8 gene of strain SHJS

The phylogenetic tree was constructed and analyzed to explore the genetic and evolutionary status of BHV strain SHJS. Phylogenetic analysis of the BHV SHJS genome showed that BHV SHJS was clustered with the K22 strain and far away from the other BHV-1 strains (Figure 3B). Due to the lack of a full genome for BHV-1.2b strains in China, we used the published BHV-1 US8 (gE) gene based on the nucleotide sequences to construct the phylogenetic tree of gE. Analysis of gE showed that BHV SHJS was displayed on a separate clade far away from the previously reported MN06 and NM14 strains isolated in China and the strains reported in the United States, India, and Australia. These results suggested that China’s newly identified BHV SHJS might be a new outbreak strain (Figure 3C).

### Phylogenetic analysis based on UL44 (gC) gene

**Figure 4:**
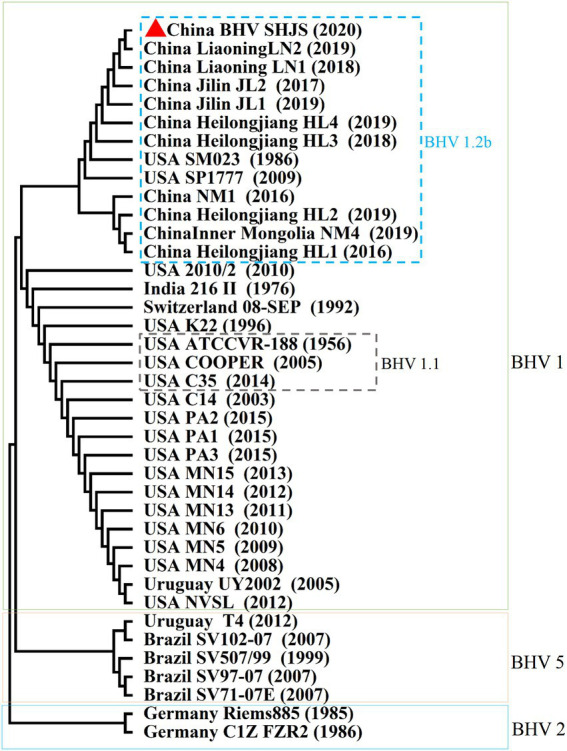
Phylogenetic tree of 451 nucleotides of partial gC gene. Thirty-eight partial nucleotide sequences (451 nucleotides) of the BHV-1 strains and partial gC gene of BHV SHJS sequences are available in GenBank and were compared using MEGA7 software. The sequences were aligned using the Clustal W method and the phylogenetic tree was generated by Maximum-likelihood analysis.

Bovine herpesvirus type 1 could be divided into BHV-1.1 and 1.2 types according to carboxy-terminal region (451 nucleotides) conserved moderately of BHV UL44 (gC) gene (Rijsewijk et al., 1999; Spilki et al., 2005; Traesel et al., 2015). BHV-1 UL44 (gC) also was suitable for phylogenetic analysis to explore evolutionary status (Chowdhury, 1995, 1997; Zhou et al., 2020). To classify the gene subtypes of BHV SHJS, the phylogenetic tree of UL44 (gC) was constructed based on 38 reference nucleotides sequence (451 nucleotides). The tree showed that isolated strains were clustered in three major clades, including BHV-1, BHV-2, and BHV-5 strains (Figure 4). BHV clade 1 was further divided into two separate lineages: lineage 1 clustering isolates of 1.2b subtypes were mainly composed of BHV SHJS (2020), Liaoning strains (2018, 2019), Jilin strains (2016, 2017, 2019), Heilongjiang strains (2018, 2019), and Inner Mongolia strains (2019) isolated in China and strains isolated in United States (1986, 2009); lineage 2 consisted of strains isolated in America, India (1976), and Switzerland (1992). BHV clade 2 consisted of strains isolated in Germany. BHV clade 5 contained strains isolated in Brazil (1999, 2007) and Uruguay (2012). In this tree, we found that BHV strain SHJS was clustered with Liaoning LN2 (2019) on one branch in the lineage close to strains isolated in China. Based on these analyses, we confirmed that BHV strain SHJS belongs to BHV-1.2 strain.

### Phylogenetic analysis of major immunogenic genes

**Figure 5:**
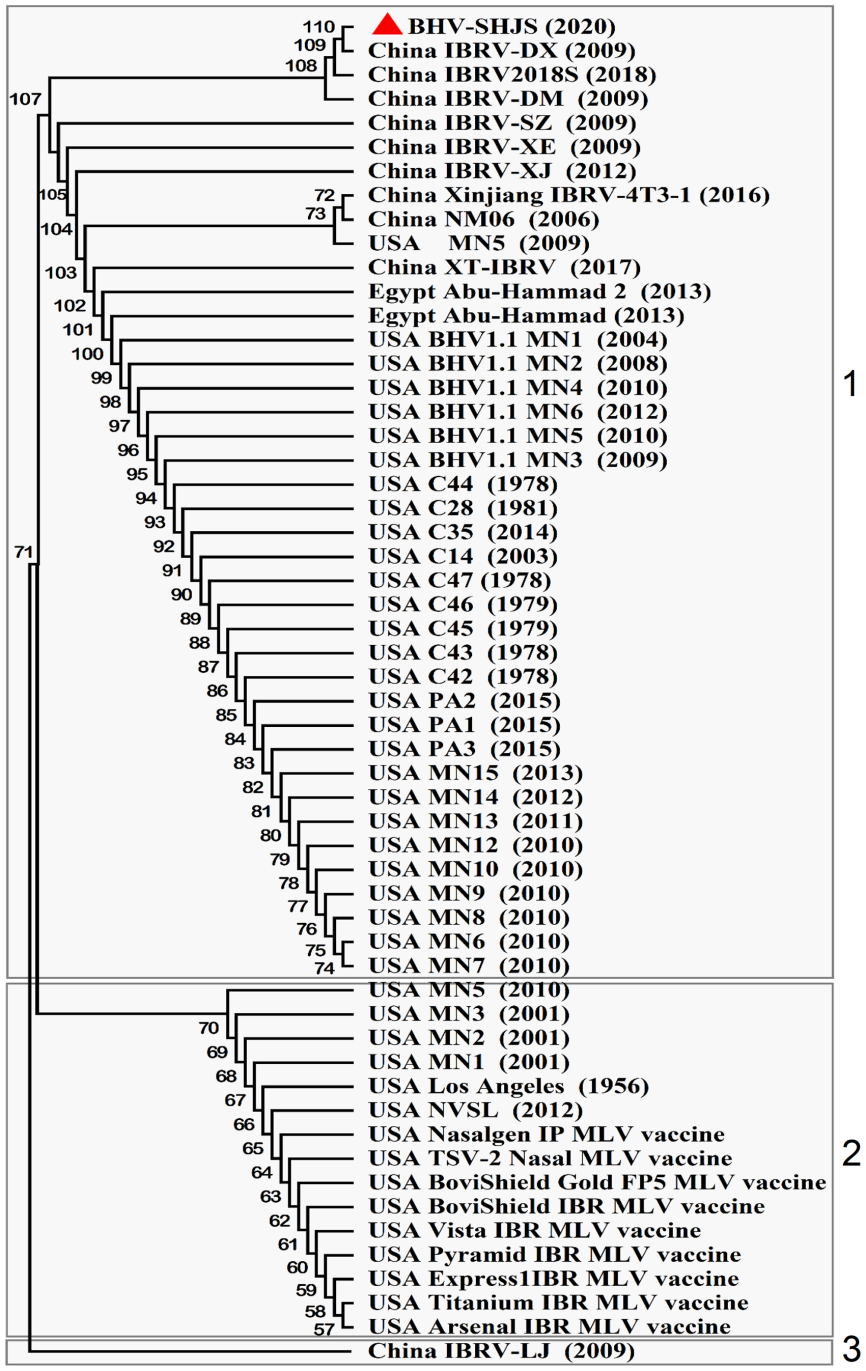
Phylogenetic tree analysis of BHV SHJS UL27 (gB) gene-based sequence. Fifty-six nucleotide sequences of BHV-1 gB are available in GenBank and were compared using MEGA7 software. The sequences of gB were aligned using the Clustal W method and the phylogenetic tree was generated by Maximum-likelihood analysis.

BHV-1 UL27(gB), UL44 (gC), and US6 (gD) encode the key immunogenic proteins that could induce neutralizing antibodies (Babiuk et al., 1987; Van Drunen Littel-van den Hurk et al., 1990; Liang et al., 1991; Yoon and Eo, 2007). To analyse whether the available BHV-1 vaccines could protect against BHV SHJS outbreaks by conducting phylogenetic tree based on the nucleotide and amino acid sequences of these genes of the available BHV strains and commercial vaccine strains. Phylogenetic analysis of gB nucleotide sequence showed that the strains were clustered in three clades (1, 2, and 3) (Figure 5). For the phylogenetic tree based on gB, BHV SHJS was closely clustered with China IBRV (2009, 2018), indicating that IBRV (2009, 2018) might be the closest ancestor of the BHV SHJS. Clade 1 consisted of BHV SHJS and other strains isolated in China, the United States, and Egypt, far from the available vaccine strains displayed in clade 2 (Figure 5). Furthermore, phylogenetic analysis of amino acid sequence of gB showed that BHV strain SHJS and American isolates were irregularly clustered in different branches with vaccine-like strain (Supplementary Figure S5). These analyses suggested that the available vaccines might not provide complete protection against BHV SHJS and might provide partial protection against other BHV-1 strains in China. This can also partially explain why BHV-1 vaccine strains only provide partial protection from the new outbreaks of BHV 1 in China or the United States.

## Discussion

Recently, infection and transmission of BHV-1 is common in cattle in China (Mo and Liu, 2017; Zhou et al., 2020). In 2018, the prevalence was 40% in cattle occurred in China (Chen et al., 2018). However, information about the prevalent of Chinese isolates is scarce. In 2020, a BHV 1.2 strain was isolated, referred to as BHV SHJS, from the nasal swabs of Holstein cows in Shanghai, China. Complete genome analyses showed that BHV SHJS has the highest nucleotide identity (99.64%) with strain SP1777 (KM258883.1) (Figure 2). The phylogenetic analysis of gC demonstrated that BHV SHJS belongs to BHV-1.2 strain. Genomic analysis presented that BHV SHJS was a new outbreak field strain in China.

We also found that mutation, deletion, or insertion occurs in some amino acids of BHV SHJS UL42 and UL46 proteins (Supplementary Figures S3B,C). BHV-1 UL46 was reported to impact viral replication (Hou et al., 2019). HSV UL46 had an important role in antiviral innate immune response (You et al., 2019). There are few in-depth studies on BHV-1 UL46 and UL42 proteins. We focused on speculating the function of UL46 and UL42-encoded proteins. We predicted the structure of proteins and showed that UL42-encoded protein consisted of 17.65% alpha helix (H), 26.72% extended β strand (E), and 55.64% loop (L) (Supplementary Figure S4A), and UL46-encoded protein consisted of 44.74% alpha helix (H), 1.8% extended β strand (E), and 53.4% loop (L) (Supplementary Figure S5A) on secondary structure. UL42-encoded protein contained Malic-M, PKS-KR domains (Supplementary Figure S4B), and UL46-encoded VP16 contains HhH1, PKS-TE, ICA69, and GDNF domains (Supplementary Figure S4C). Insertions of BHV SHJS UL42 occurred in the predicted PKS-KR domain, which was associated with bacterial polyketide synthases and catalyzes. Deletions and mutations of BHV SHJS UL46 occurred in an Islet cell autoantigen ICA69 domain and GDNF domain which is a potent survival factor for sympathetic, sensory, and central nervous system neurons (each functional domain description is derived from the database on prediction). Based on these predictions, we speculated that the functions of these two proteins might be similar to these functional domains. However, mutation, deletion, or insertion of these domains might influence protein functions.

Currently, commercial inactivated or attenuated live vaccines and recombinant subunit vaccines are used to immunize cattle to gain protection against BHV-1 (Cowley et al., 2011; Biswas et al., 2013; Fulton et al., 2013; Gonçalves et al., 2021). However, these vaccines only partially protect against BHV-1, as observed in China or the United States (O'Toole et al., 2012). Phylogenetic analyses of immunogenic genes (gB) revealed that BHV SHJS and some other Chinese outbreaks and some American vaccine strains were displayed on different branches (Figure 5). Based on these analyses, we speculate that the available vaccines in China might not protect BHV SHJS and might only provide partial protection against other BHV-1 strains in China. This also partially explains why BHV-1 vaccine strains only provide partial protection from the new outbreaks of BHV 1.2 in China or the United States.

It has been reported that homologous recombination often occurs in the herpesvirus species: Pseudorabies virus (PRV), Varicella-zoster virus, and herpes simplex virus type 1 (HSV-1) (Kintner et al., 1995; Schynts et al., 2003; Thiry et al., 2005; Norberg et al., 2015; Bo et al., 2021). Thus, there was a huge responsibility placed on virus infection prevention. Recombination events between BHV-1 and BHV-5 are also reported (Meurens et al., 2004). Recombination of live-attenuated BHV-1 was first documented in Australia in 2008 (Lee et al., 2012). Whether BHV SHJS isolated also occurred recombination after vaccination, we attempted and performed a recombination prediction analysis with nine commercial vaccine strains. In prediction, we found that BHV SHJS has a potential recombinant region in the genome of BHV SHJS (Supplementary Figure S6). We speculated that BHV SHJS might occur recombination with vaccine in the future prevention, which needs more evidence to prove that a recombination event occurs in the future.

## Data availability statement

The data presented in the study are deposited in the GenBank repository, accession number OP035381, which can be found at: https://www.ncbi.nlm.nih.gov/nuccore/OP035381.

## Author contributions

WG, Y-SJ, and HC conceived and designed the experiments. WG and JX performed the experiments. JL and WG analyzed the data. HC and Y-SJ helped with reagents, materials and analytical tool. WG, HC, and JL wrote the manuscript. All authors contributed to the article and approved the submitted version.

## Funding

This experiment was supported by the National Nature Science Foundation of China (grant numbers 32170161 and U19A2039).

## Conflict of interest

The authors declare that the research was conducted in the absence of any commercial or financial relationships that could be construed as a potential conflict of interest.

## Publisher’s note

All claims expressed in this article are solely those of the authors and do not necessarily represent those of their affiliated organizations, or those of the publisher, the editors and the reviewers. Any product that may be evaluated in this article, or claim that may be made by its manufacturer, is not guaranteed or endorsed by the publisher.

## Footnotes

^1^ https://www.ebi.ac.uk/Tools/msa/clustalo/

^2^ https://open.predictprotein.org/

^3^ https://smart.Embl.de/
