# Supplementary material for: The full-genome characterization and phylogenetic analysis of bovine herpesvirus type 1.2 isolated in China
Source: Front Microbiol. 2022 Nov 1;13:1033008. doi: 10.3389/fmicb.2022.1033008 (PMC9664903; doi:10.3389/fmicb.2022.1033008)
Supplement: Supplementary file 1 [file Data_Sheet_1.docx]

**Supplementary information**

**The Full-Genome Characterization and Phylogenetic Analysis of Bovine Herpesvirus Type 1.2 Isolated in China**

**Weiqiang Guo ^1^, Jia Xie ^2^,** **Jingyi Liu ^2^****, Hongjun Chen** **^2^,** **Yong-Sam Jung ^1*^**

^1^ MOE Joint International Research Laboratory of Animal Health and Food Safety, College of Veterinary Medicine,

Nanjing Agricultural University, Nanjing, Jiangsu, China

^2^ Shanghai Veterinary Research Institute, Chinese Academy of Agricultural Sciences, Shanghai, China

*Correspondence

**Yong-Sam Jung,** MOE Joint International Research Laboratory of Animal Health and Food Safety, College of Veterinary Medicine, Nanjing Agricultural University, Nanjing, Jiangsu, China. E-mail: [ysjung@njau.edu.cn](mailto:ysjung@njau.edu.cn).

**TABLE S1 PCR primer sequences**

| **Name** | **Sequence** |
| --- | --- |
| BHV-UL48-F | 5′-CCGGAATTCATGAGCGGGCGCATAAAA-3′ |
| BHV-UL48-R | 5′-CGCTCGAGGAAGTCCAGCAGCTGGTT-3′ |
| BHV-UL49-F | 5′-GGAATTCATGGCCCGGTTCCACAGG-3′ |
| BHV-UL49-R | 5′-CCCAAGCTTGCCGCGAAGGCGGCTTTC-3′ |
| BHV-US7-F | 5′-CCGGAATTCATGCGGTGCCTGTTGCTCTGG-3′ |
| BHV-US7-R | 5′-GCTCTAGATTATTCTTCGCTGATGGTGGCGAGGGG-3′ |
| BHV-US8-F | 5′-CCGGAATTCATGCAACCCACCGCGCCGCCC-3′ |
| BHV-US8-R | 5′-GCTCTAGACTAGCGGAGGATGGACTTGAGTCGCGC-3′ |


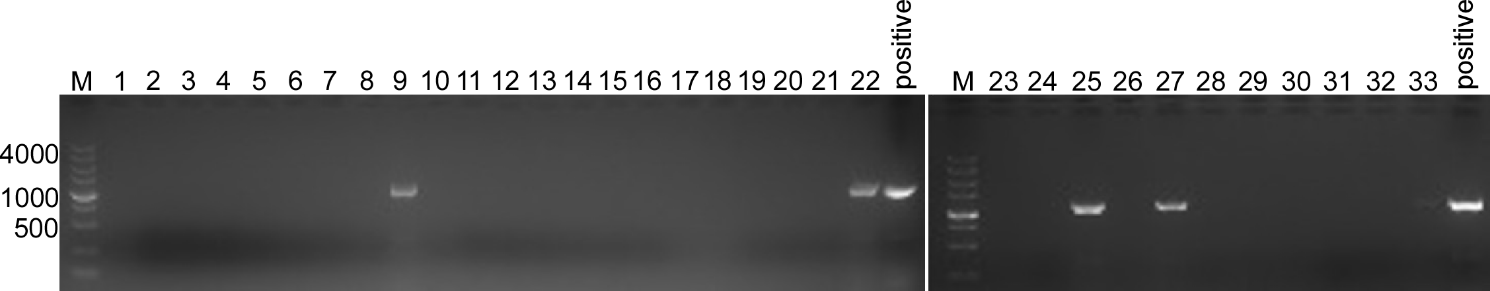


**FIGURE S1. Results of PCR amplification and alignment products with BHV conserved genes.**

Fragments were obtained successfully by PCR amplification from DNA extraction of the Thirty-three nasal swabs samples (lane No.1 to 33) using specific primers for BHV-1 US7. Fragment of positive control was amplified from genomic DNA of BHV-1 strain Bartha, which was marked with positive label in lanes. The PCR fragment of BHV-1 US7 was aligned with the reference strain (KM258882.1).


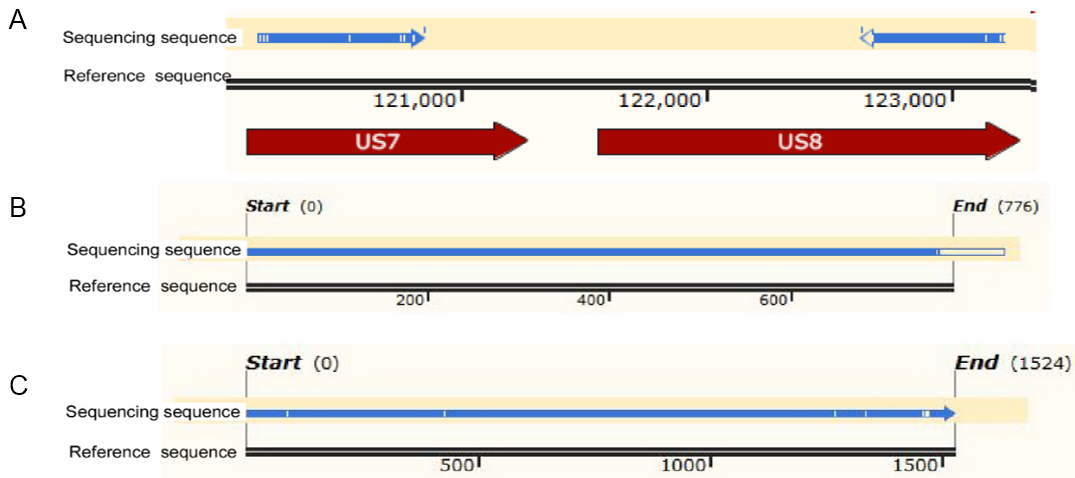


**FIGURE S2. Alignment results of sequence of PCR amplified products with BHV-1 conserved genes.**

Sequence alignments of PCR products have high similarity to BHV-1 US7/US8 (3156bp) (A), UL48 (1524bp) (B), and UL49 (776bp) (C).


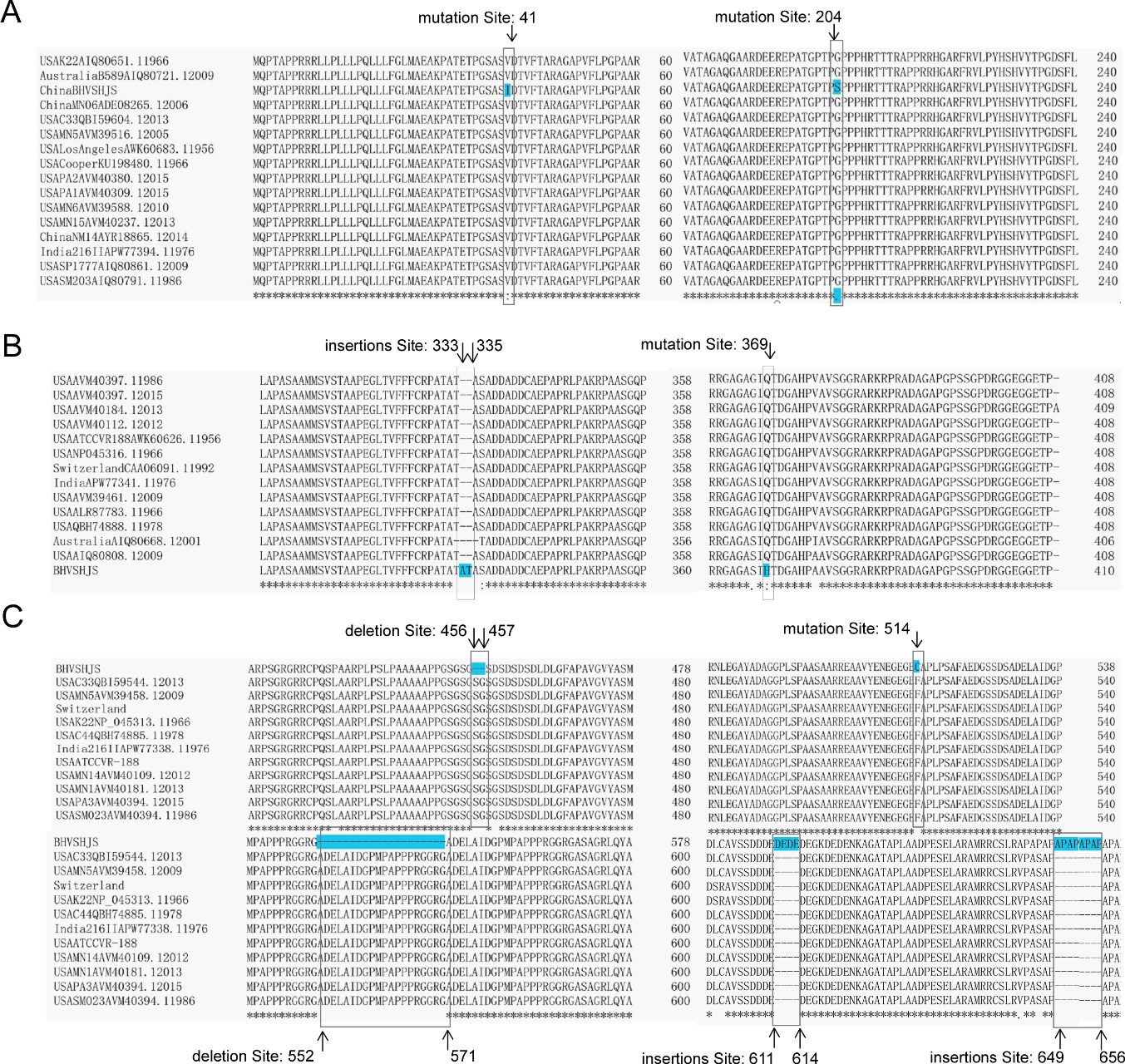


**FIGURE S3. Alignment analysis of amino acid sequences of BHV SHJS US8, UL42 and UL46.**

(A) Alignment of gE sequence showed that have two mutation sites, one site at 41 Val (Val, V) mutated to Ile (Ile, I), the other mutation site at 204 G (Gly, G) mutated to S (Ser, S).

(B) Alignment of UL42 sequence showed two insertions at 333 Ser (S) and 334 Ala (A), and one mutation site at 369 H (His) mutated to Q (Gln).

(C) Alignment of UL46 sequence presented two deletion sites at 456 to 457, 552 to 571 and 648 to 653, two insertion regions located at 611 Glu (E) to 614 Asp (D) and 649 Pro (P) to 656 Ala (A), and one mutation site at 514 F (Phe) mutated to C (Cys).

**
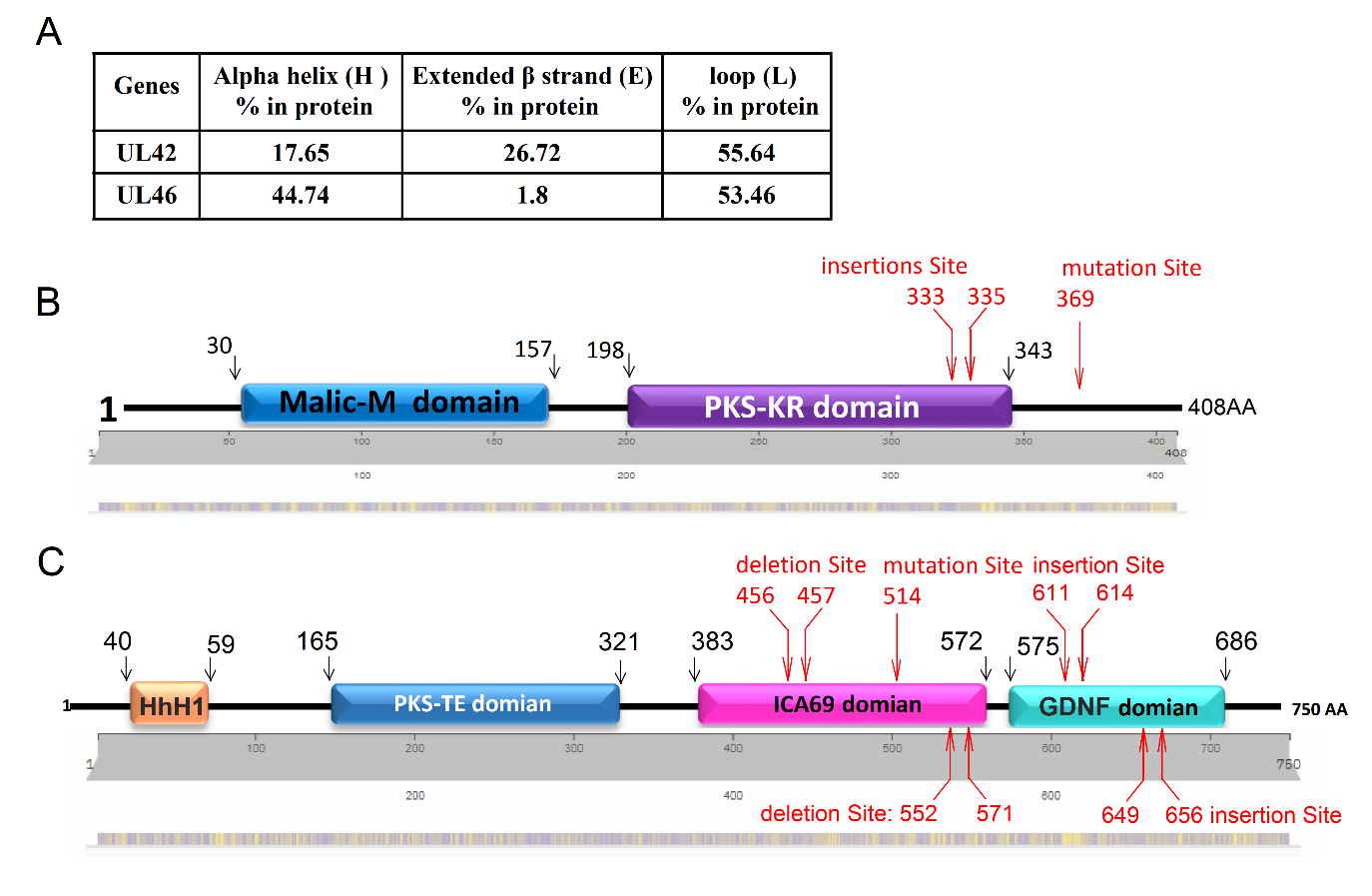
**

**FIGURE S4. Structure of UL42 and UL46 by website prediction.**

(A) UL42 and UL46 encoded protein secondary structure was predicted in website( https://predictprotein. org/).

(B) UL42 encoded protein was predicted by domain website (<http://smart.embl.de/>). Result shown that it contains Malic-M, PKS-KR domains. Insertions of BHV SHJS UL42 occurs in the predicted PKS-KR domain.

(C) UL46 encoded protein was predicted by domain website (<http://smart.embl.de/>). Result shown that it contains HhH1, PKS-TE, ICA69 and GDNF domains. Deletions and mutations of BHV SHJS UL46 occurs in an Islet cell autoantigen ICA69 domain and GDNF domain.

**
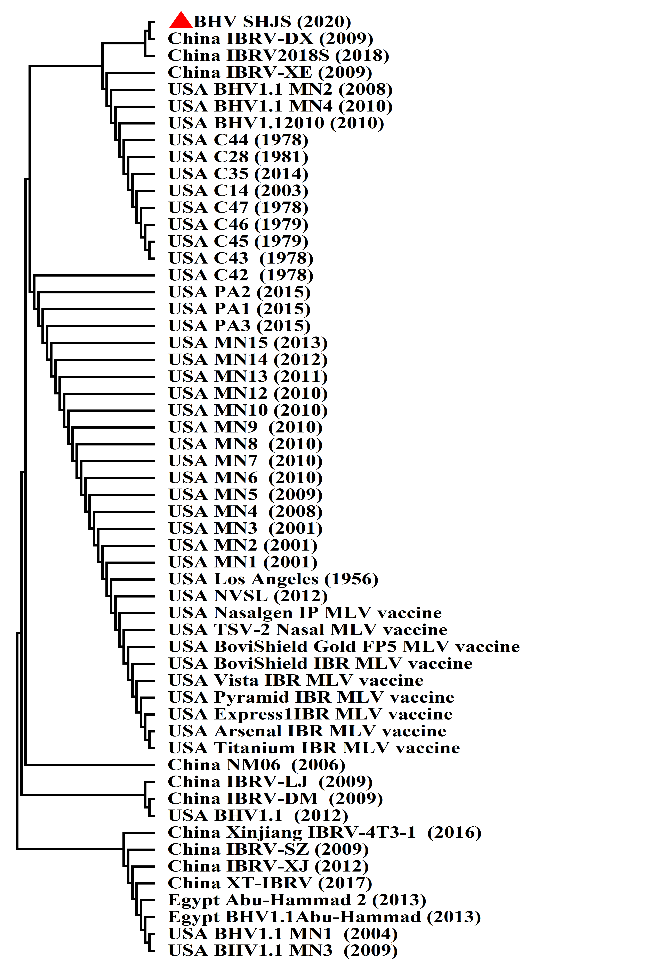
**

**FIGURE S5. Maximum-likelihood analysis of BHV SHJS UL27 (gB) gene based amino acid sequence.**

Fifty-six sequences of BHV SHJS UL27 gene are available in GenBank and were compared using MEGA7 software. The amino acid sequences were aligned using the Clustal W method.

**
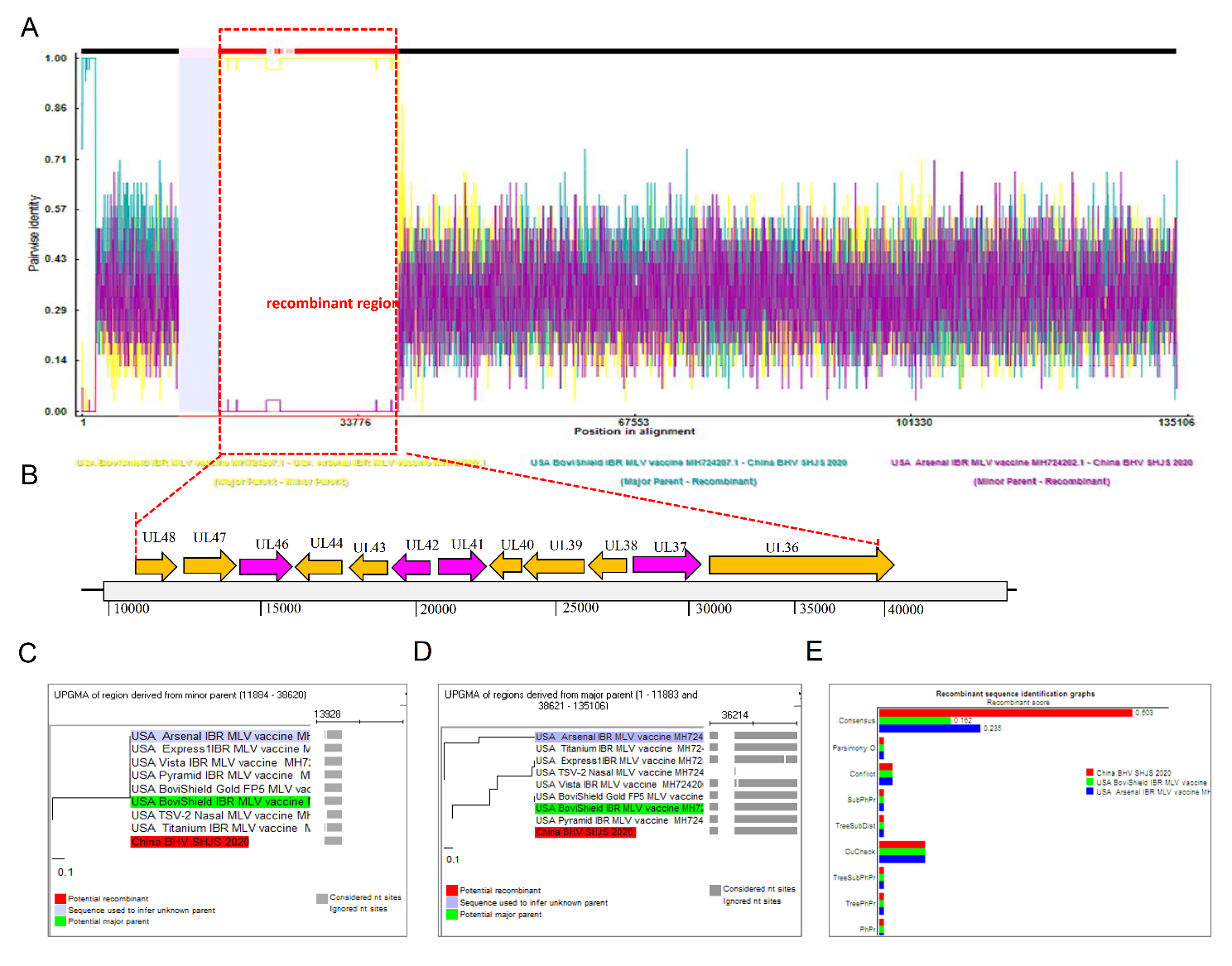
**

**FIGURE S6. The** **Recombinant prediction analysis of BHV SHJS and vaccine strain.**

RDP (version 3.5.1) software was used to recombination analysis based on published nine BHV-1 vaccine strain genome in the NCBI database. In prediction analysis, we found that BHV SHJS has a potential recombinant region (A) in the genome at 1,1884 to 38620 nt. Predicted recombination regions contained ORFs by alignment using the Clustal Omega website. Recombination might occur in UL46, UL42, UL41, and UL37 by prediction and are marked in pink. Non-recombination ORFs are marked in orange (B). The tree analysis shown that parents of BHV SHJS were BoviShield IBR MLV and Arsenal IBR MLV vaccine strain on evolutionary relationship of recombinant evens (C, D). Recombination score showed that the score of BHV SHJS evens was 60.3 % (E). It suggested that a high confidence in recombination prediction analysis. (A) Predicted recombinant region of BHV SHJS with vaccine strain. (B) The specific exchange points and representative ORFs parts in recombination regions. (C) The tree was constructed based on the sequence of the recombination regions sequences. (D) The tree was constructed based on non-recombinant regions sequences. (E) Summary of recombination event score on consistency.

**Table S2 The position information of ORF of genes in BHV SHJS genome**

**
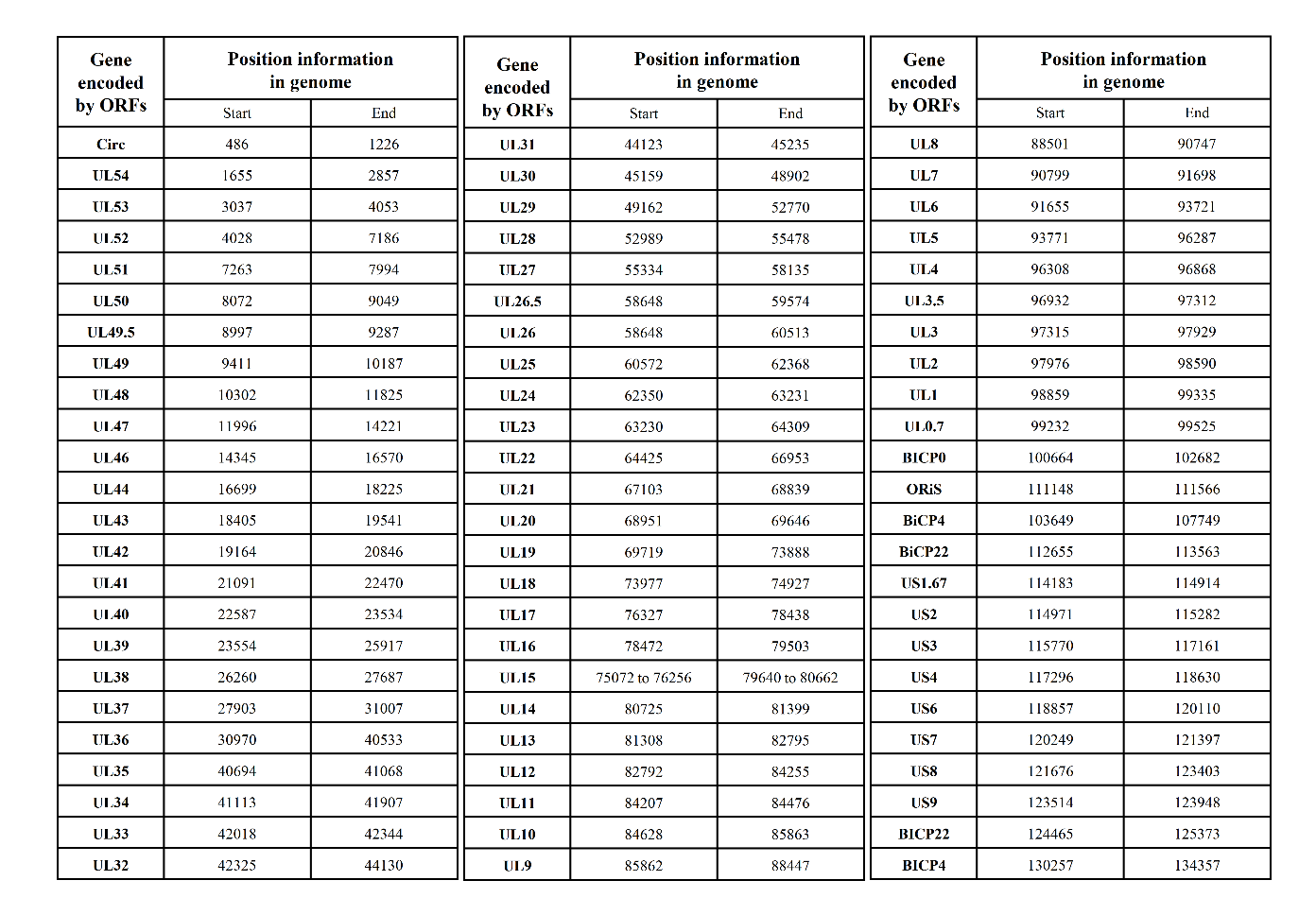
**
